# Thyroid hormone acts independently of the thyroid hormone receptor beta in hepatocytes to improve systemic insulin sensitivity

**DOI:** 10.1097/HC9.0000000000000937

**Published:** 2026-05-08

**Authors:** Anne H. van der Spek, Megan J. Ritter, Izuki Amano, Lorraine Soares de Oliveira, Joseph E. Kaserman, Isabella Salguero Cespedes, Taylor Vierling, Andrew A. Wilson, Anthony N. Hollenberg

**Affiliations:** 1 Department of Endocrinology, Amsterdam UMC; Amsterdam Gastroenterology, Endocrinology & Metabolism, Amsterdam, the Netherlands; 2 Joan and Sanford I. Weill Department of Medicine, Division of Endocrinology, Diabetes, and Metabolism, New York, New York, USA; 3 Department of Medicine, Section of Endocrinology, Diabetes, Nutrition, and Weight Management, Boston, Massachusetts, USA; 4 Department of Integrative Physiology, Gunma University Graduate School of Medicine, Maebashi, Japan; 5 Department of Medicine, Section of Pulmonary, Allergy, Sleep & Critical Care Medicine, Boston, Massachusetts, USA; 6 Center for Regenerative Medicine (CReM), Boston University and Boston Medical Center, Boston, Massachusetts, USA

**Keywords:** cirrhosis, endocrinology, liver disease, MASH, MASLD

## Abstract

**Background:**

Insulin resistance plays a key role in the development of type 2 diabetes and predates the development of frank hyperglycemia. Thyroid hormone (TH) signaling plays a critical role in glucose homeostasis, as both hyperthyroidism and hypothyroidism have been linked to the development of insulin resistance and diabetes. The mechanism behind the effects of TH action on insulin sensitivity is incompletely understood, but the liver is thought to play a key role. Indeed, resmetirom, a selective thyroid hormone receptor beta (THRβ) agonist, has recently been approved for treatment of liver fibrosis, and more THRβ agonists are currently in phase 2–3 clinical trials for use in metabolic dysfunction–associated fatty liver disease. As insulin resistance is closely associated with this disease, it is crucial that we understand the role of hepatic THRβ in glucose homeostasis. Thus, we hypothesized that TH, acting via the THRβ, is a key regulator of hepatic glucose metabolism.

**Methods:**

In wild-type (WT) and liver-specific THRβ knock-out (L-TRBKO) mice we analyzed the effect of changes in thyroid status and diet on glucose homeostasis and insulin signaling. Mice were assessed under basal conditions on a chow fed diet, under hypothyroid conditions using a propylthiouracil/low iodine diet with and without T3 treatment and following a high-fat diet. We measured glucose tolerance, hepatic insulin signaling, liver histology, energy expenditure and skeletal muscle metabolism. In high-fat diet fed WT and L-TRBKO mice we addidionally analyzed the effect of a single i.p. injection of T3. Finally we studied insulin signaling in human induced pluripotent stem cells differentiated to hepatocytes (iHeps) both with and without THRβ expression.

**Results:**

In contrast to our hypothesis, we found that insulin signaling in mice was not impacted by the selective deletion of THRβ only in hepatocytes. Both WT and L-TRBKO mice have similar glucose homeostasis under basal conditions and developed hyperglycemia on a high-fat diet. Further, a single dose of T3 administered to high-fat diet fed insulin-resistant mice improves insulin sensitivity to the levels of control chow-fed mice in both WT and L-TRBKO male mice. This single dose of T3 also increased glucose transporter expression in skeletal muscle. In iHeps, THRβ1 was not required to activate insulin signaling, and T3 treatment did not affect insulin signaling.

**Conclusion:**

T3 signaling impacts glucose homeostasis independently of its actions through the THRβ1 in hepatocytes in both a murine and human model.

## Graphical abstract

**Figure:**
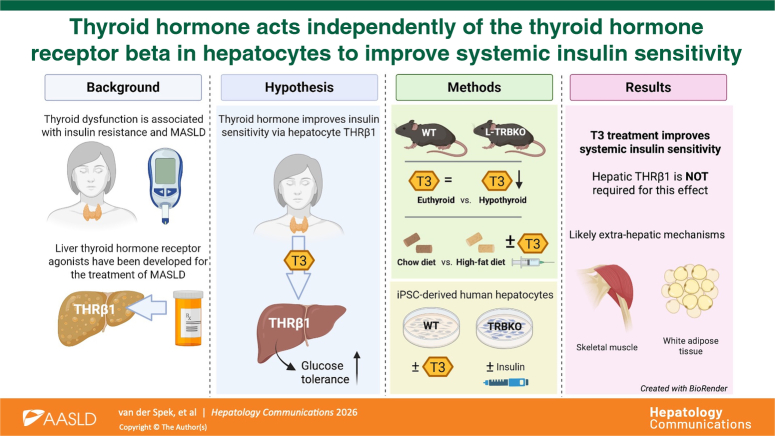

## INTRODUCTION

Metabolic diseases, including type 2 diabetes and metabolic dysfunction- associated steatotic fatty liver disease (MASLD, formerly non-alcoholic fatty liver disease; NAFLD), continue to rise worldwide.^1^^,^^2^ In 2021, there were an estimated 6.7 million deaths globally from diabetes, and the prevalence of metabolic diseases and insulin resistance (IR) is estimated to be between 20% and 40% and rising across various populations.^3^^–^^5^ IR, an inability of insulin to stimulate appropriate glucose disposal, may be a precursor to the development of type 2 diabetes and MASLD.^6^^,^^7^ IR is often asymptomatic and remains undetected while end-organ damage accumulates.^8^ IR also inhibits appropriate hepatic glucose production, worsening hyperinsulinemia, contributing to an excess of hepatic triglycerides and the development of MASLD.^6^^,^^9^^,^^10^ Targeting key pathways of metabolic diseases, including MASLD and IR, continues to be an area of clinical interest.

Thyroid hormones (TH) are well-established regulators of energy and glucose metabolism, and thyroid dysfunction has been associated with the development of IR.^11^ In the liver, TH signaling acts on pathways that regulate cholesterol accumulation, lipid metabolism (fatty acid beta-oxidation and de novo lipogenesis), and glucose metabolism.^12^ Given these effects on hepatic lipid metabolism, thyroid hormone receptor (THR) agonists—such as resmetirom—have been developed for the treatment of MASLD and metabolic dysfunction–associated steatohepatitis (MASH).^13^^–^^15^ The THRs, which mediate TH action, are nuclear receptors with 2 main isoforms, THRα and THRβ. Both act as ligand-inducible transcription factors, such that they possess separate activities in the presence or absence of triiodothyronine (T3). The predominant isoform expressed in the liver is THRβ1, the primary target of the newly developed class of liver TH agonists, including resmetirom.^16^ With the introduction of THR agonists into clinical practice, it is increasingly important to understand how hepatic THRβ activation affects other metabolic components of liver disease. IR, a hallmark of MASLD and MASH, is one such aspect.^17^ Indeed, the literature suggests that TH plays a pivotal role in hepatic glucose metabolism.^17^

At the molecular level, TH induces hepatic gluconeogenesis by activating PEPCK and inhibits insulin signaling by decreasing Akt phosphorylation via a pathway mediated by forkhead box protein O1 (FOXO1) that requires THRβ.^18^ These mechanistic studies suggest a key role for T3 and hepatic THRβ in glucose and insulin metabolism. However, in vivo murine and human studies find varying effects of alterations in TH signaling on insulin sensitivity. Serum TH concentrations are positively associated with higher fasting glucose and increased IR in large population-based cohorts.^19^ In patients with hyperthyroidism, insulin and glucose levels are elevated compared with euthyroid controls, and both glucose and insulin levels improve with normalization of TH levels.^20^^,^^21^ Similarly, in Zucker fatty rats, acute treatment with T3 leads to hyperglycemia.^22^ Interestingly, the use of THRβ agonists yields varying results on insulin sensitivity, with both improved and diminished insulin sensitivity reported in animal studies, and changes in glucose metabolism seen in clinical trials varies.^13^^,^^23^^,^^24^

Hypothyroidism, defined by low levels of TH, is also associated with IR. Patients with hypothyroidism have higher rates of hepatic triglycerides and MASLD rates.^16^ Metabolic impairments have been found to be associated with even mild disturbances of thyroid function.^11^ In patients with Resistance to Thyroid Hormone Beta (RTH-Beta), where THRβ signaling is suppressed, impaired insulin sensitivity has been described.^25^^,^^26^ In contrast, other authors have found unaffected insulin sensitivity in RTH-Beta patients.^27^ Thus, despite some clear clinical effects on glucose metabolism and insulin sensitivity, the systemic regulation of glucose by TH and the THR is inadequately and incompletely understood.

Here, we sought to elucidate the role of hepatic THRβ1 in IR using mouse and human models. We hypothesized that T3 acting via the THRβ1 is a key regulator of hepatic glucose metabolism and insulin sensitivity. Surprisingly, we found that hepatic THRβ1 did not affect systemic glucose tolerance or hepatocyte insulin signaling. Both high-fat diet and hypothyroidism induced systemic IR in mice, which was improved by T3 treatment, independently of THRβ1 in hepatocytes. The improvement in IR was in part mediated by an increase in GLUT4 in skeletal muscle.

## METHODS

### Animals

All animal experiments were conducted in compliance with relevant institutional and (inter)national guidelines and regulations. Experimental protocols were approved by the Weill Cornell Medicine Institutional Animal Care and Use Committee. Mice were housed in a 12-hour light/dark cycle and supplied with food ad libitum. Mice were euthanized by CO_2_ asphyxiation followed by cardiac puncture in accordance with accepted veterinary standards. Tissues were rapidly collected and frozen in liquid nitrogen before storage at −80 °C, unless collected for histology. Liver-specific TRβ1KO mice were generated as previously described.^28^ Briefly, *Thrb* floxed mice were crossed to mice that express Cre recombinase under the control of albumin promoter, which produced Cre expression in hepatocytes only [(B6.Cg-Tg(Alb-cre)21Mgn/J), JAX Cat#003574]. *TR*β*f/f* mice were crossed with *TR*β*f/f Alb-Cre/−* mice, which generated the littermate controls *TR*β*f/f* and *L-TRβ1KO*. *Thrb* floxed mice were obtained from the Gauthier laboratory, Institut de Génomique Fonctionnelle de Lyon Université de Lyon, Lyon, France.^29^ All mice were maintained on a C57BL/6J genetic background.

### PTU low iodine treatment

**FIGURE 1:**
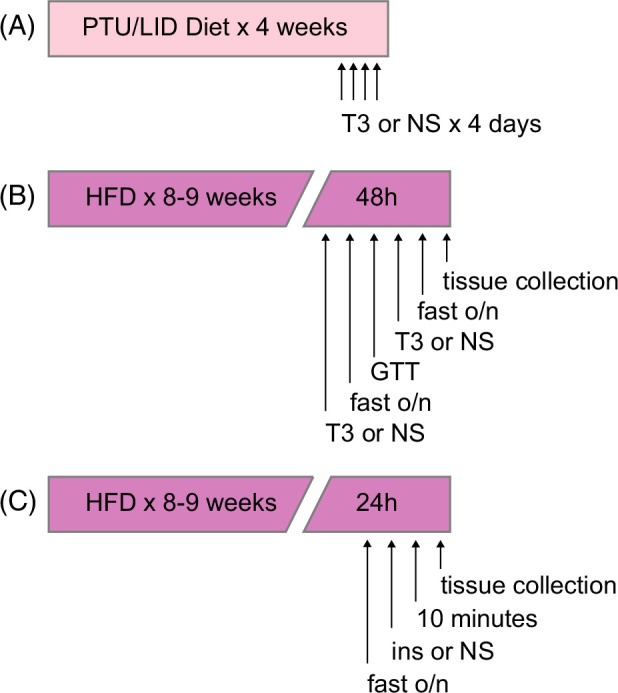
Treatment timelines. (A) Mice were placed on a PTU/LID diet for 4 weeks, and during the last 4 days, they were treated with T3 i.p. (B) Mice were placed on an HFD for 8–9 weeks, followed by T3 or normal saline (NS) i.p. before a GTT. (C) Mice were placed on an HFD for 8–9 weeks, followed by insulin i.p. or NS, 10 minutes after which they were euthanized and tissues collected. Abbreviations: GTT, glucose tolerance test; HFD, high-fat diet; NS, normal saline; PTU/LID, propylthiouracil/low iodine diet; T3, triiodothyronine.

Eight- to ten-week-old male mice were maintained on a standard chow diet or put on propylthiouracil/low iodine (PTU/LID) diet for 4 weeks [0.15% propylthiouracil, low iodine (TD.95125; Envigo)] to induce hypothyroidism.^28^ The PTU/LID +T3 groups received daily (i.p.) injections of T3 (2 μg/100 g body weight) for the last 4 days of the experiment.^28^ The PTU/LID—T3 group received i.p. injections with sterile saline. On day 28 of the experiments, mice were euthanized in the fed condition. Blood and tissues were collected as described above (Figure 1A).

### High-fat diet

Six- to eight-week-old male and female mice were maintained on a standard chow diet or put on a high-fat diet (HFD) for 8–9 weeks (0.3% kcal from fat, 21.4% kcal from carbohydrates, 18.3% kcal from protein; TD.06414; Envigo) to induce obesity.

Before euthanasia, mice were fasted overnight, and glucose was determined using a flash glucometer with blood collected from the tail vein. Mice were euthanized in the fasted condition. A subset of animals received i.p. insulin injections (1 IU/kg body weight, human insulin; Sigma) 10 minutes before euthanasia. Blood and tissues were collected as described above (Figures 1B, C).

### T3 treatment

Mice on HFD were injected intraperitoneally with T3 (2 μg/100 g body weight) or sterile saline. Mice received i.p. T3 injections for 2 consecutive days at ~4 PM before being fasted overnight and euthanized in the fasted state. Mice undergoing an i.p. glucose tolerance test (ipGTT) underwent this the day after the first injection of T3. Following the ipGTT, mice were refed and received their second injection with T3 (2 μg/100 g body weight) or sterile saline. They were then fasted overnight and euthanized in the fasted state (Figure 1B).

### Intraperitoneal glucose tolerance test

Glucose was dissolved in sterile saline, and 2 g/kg body weight glucose was administered by i.p. injection after an overnight fast. Fasting blood glucose levels were checked right before glucose administration from tail cuts using a OneTouch glucometer. Glucose levels were checked at 15-, 30-, 60-, 90-, and 120-minutes after initial dosing.

### Metabolic phenotyping

Comprehensive metabolic monitoring was performed on male mice using a Promethion Metabolic Screening System (Promethion High-Definition Multiplexed Respirometry System for Mice; Sable Systems International, North Las Vegas, NV, USA) as previously described.^30^ Mice were individually housed in metabolic cages within temperature- and light-controlled enclosures (22 °C, 12 h light/dark cycle) (DB034-LT Laboratory Incubator; Darwin Chambers Company, St Louis, MO, USA), on pine chip bedding, without nestlets, with ad libitum food and acidified water, and acclimatized for 48 hours. Mice underwent 24-hour metabolic cage monitoring before removal for body composition analysis and i.p. T3 injection, then they were returned for another 24 hours of monitoring, followed by repeat body composition analysis.

Energy expenditure was assessed via indirect calorimetry and calculated using the Weir equation (3.941 kcal/L × VO_2_ + 1.106 kcal/L × VCO_2_).^31^ Respirometry values, dwell time, baseline cage sampling frequency, and rates of oxygen and carbon dioxide production were determined as previously described.^30^ Food and water intake, and body mass were assessed gravimetrically. Distance traveled (activity) was determined by XY position displacements that are represented by beam breaks (PedMeters and AllMeters). Body composition was analyzed using EchoMRI (EchoMRI Body Composition Analyzer).

### Maintenance of inducible pluripotent stem cells (iPSCs) and generation of iPSC-derived hepatocytes

The human iPSC line BU3-10-Cr2 was obtained from Center for Regenerative Medicine, Boston University and previously published.^32^ The iPSC parental and gene-edited (“TRBKO”) lines were generated and cultured as previously published.^33^ Detailed iPSC derivation and culture protocols are available at: https://crem.bu.edu/cores-protocols/. iPSC-directed differentiation to hepatocytes was performed using our previously published protocol.^33^^–^^35^

### iHep T3 and insulin treatment

Day 25 iPSC-derived hepatocytes (iHeps) were incubated for 24 hours in washout media (75% IMDM, 25% Hams F12, 50 μg/mL ascorbic acid, 1X primocin, and 0.05% of BSA) to prevent activation of Akt by other components of the medium. T3 100 nM or phosphate-buffered saline (PBS) vehicle control was added to the medium and incubated for 24 hours. Following T3 treatment, iHeps were treated with 10 nM of human insulin or PBS vehicle control (Sigma-Aldrich) for 10 minutes before cell harvest.

### RNA, cDNA, and real-time qPCR

RNA was extracted from liver, skeletal muscle, and white adipose tissue using RNA STAT-60 (Fisher Scientific), and 500 ng of RNA was reverse transcribed into cDNA using the SuperScript VILO kit (Invitrogen). RNA concentration and purity were determined using a Thermo Scientific NanoDrop spectrophotometer. Samples with low concentration or suboptimal A260/280 and A260/230 ratios were excluded from downstream cDNA synthesis and qPCR.

Real-time, quantitative PCR (qPCR) was performed in duplicate for all samples using predesigned TaqMan probes and TaqMan Universal Master Mix (Applied Biosystems), and the QuantStudio 6 Pro system (Thermo Fisher) was used. Relative mRNA levels were calculated using standard-curve methods and normalized to the expression level of Eukaryotic 18S rRNA Endogenous Control (VIC/MGB Probe— Applied Biosystems).

### Histology

Liver was fixed in 10% formalin solution for 48 hours, and paraffin-embedded sections were used for routine H and E staining.

### Western blot

*For mouse liver*: 50 mg of liver was homogenized in 1 mL of cell lysis buffer, 1X proteinase inhibitor, and 1X PMSF. Samples were sonicated on ice and then spun at 13,200 RPMs for 15 minutes at 4°C before being stored at −80°C. Protein concentrations were determined using a BCA reagent (Pierce). Western blots were performed using 20 μg of protein.

*For iHep samples*: Cells were harvested in cell lysis buffer (Cell Signaling Technology) with 20 μg/mL aPMSF, 1x protease and 1x phosphatase inhibitors (Roche). Samples were sonicated on ice and then spun at 14,000*g* for 10 minutes at 4°C before being stored at −80°C. Protein concentrations were determined using a BCA reagent (Pierce).

Protein lysates were resolved in 4%–12% Bis-Tris gel (Invitrogen) and transferred to a nitrocellulose membrane. Blots were probed using rabbit polyclonal anti-p-Akt Ser473 antibody (Cell Signaling Technology #9271, 1:2500) or rabbit polyclonal anti-Akt (Cell Signaling Technology #9272, 1:2500) overnight at 4°C with mouse monoclonal anti-beta-actin antibody (MA191399; Fisher Scientific; 1:5000). Goat anti-rabbit Alexa Fluor Plus 680 (A32734; Invitrogen) and goat anti-mouse Alexa Fluor Plus 488 (A32723; Invitrogen) were used as secondary antibodies and incubated at room temperature for 60 minutes. Images were captured on ChemiDoc Touch MP (Bio-Rad).

### TSH and thyroid hormone measurements

Total plasma T4 levels were measured using AccuDiag ELISA Kit (3149-18; Cortez Diagnostics, Inc.). Thyroid-stimulating hormone was measured in plasma using Milliplex MAP Mouse Pituitary Magnetic Bead Panel (MPTMAG-49K; MilliporeSigma).

### Insulin ELISA

Serum insulin concentrations were measured using the Mouse Insulin ELISA Kit (EMINS; Invitrogen) according to the manufacturer’s instructions.

### Statistical analysis

Statistical analysis was performed using GraphPad Prism, 9.3.0. All figures show representative data from independent biological replicates. The differences in mRNA expression and circulating hormone levels were analyzed by the Student *t* test when comparing two groups and by 2-way ANOVA with repeated measures, followed by post-hoc Bonferroni multiple comparisons test when comparing different treatments and genotypes within 1 experiment. Body weight, body composition, blood glucose, metabolic phenotyping data, quantified western blots, and GTTs were analyzed by 2-way ANOVA with repeated measures followed by post-hoc Bonferroni multiple comparisons. Fasting glucose and AUC were analyzed by 1-way ANOVA followed by post-hoc Tukey’s multiple comparisons test when comparing 3 groups or by 2-way ANOVA with repeated measures followed by post-hoc Bonferroni multiple comparisons test when comparing different treatments and genotypes within 1 experiment. Data are expressed as mean ± SEM, and *p*-values <0.05 were considered statistically significant.

## RESULTS

### Thyroid function and glucose tolerance are the same in mice with and without hepatic Thrβ1

**FIGURE 2:**
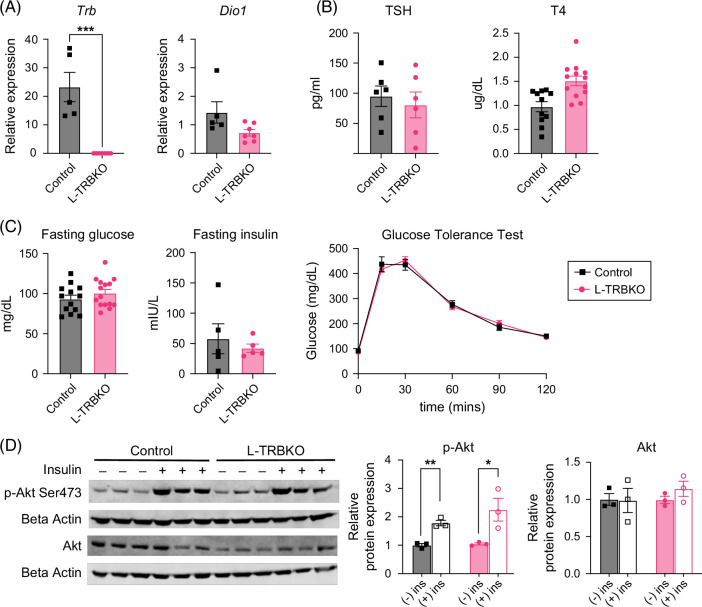
Loss of thyroid hormone receptor β1 in the liver does not affect systemic thyroid status or glucose tolerance. (A) Liver mRNA expression levels of *Thrb1* and *Dio1*, a positively regulated TH target gene in L-TRBKO mice and controls. (B) Circulating thyroid-stimulating hormone (TSH, or thyrotropin) and T4 in L-TRBKO mice and controls. (C) Fasting blood glucose and blood insulin levels in L-TRBKO mice and controls. (D) Glucose tolerance test (GTT) using i.p. injection with 2 g glucose/kg body weight in L-TRBKO mice and controls after an overnight fast. (D) L-TRBKO and control mice were injected intraperitoneally with 1 IU/kg human insulin 10 minutes before euthanasia. Phosphorylation of Akt in the liver was measured using a western blot to assess activation of the insulin signaling pathway. Data are shown as mean ± SEM. *p<0.05, **p<0.01, ***p<0.001, N=5–13 per group for qPCR, thyroid function, and GTT. Abbreviations: L-TRBKO, liver-specific THRβ knock-out; mRNA, messenger RNA.

To establish the role of hepatic Thrβ1 on glucose metabolism and insulin signaling in the euthyroid state, we compared control mice with mice that lack *Thr*β*1* expression in hepatocytes (L-TRBKO mice). Male L-TRBKO mice expressed no *Thr*β in the liver while expressing *Dio1*, a key positive TH gene target, at levels similar to controls (Figure 2A), consistent with previous observations where the lack of Thrβ does not alter TH target gene expression in the euthyroid state.^28^^,^^33^ We confirmed this change was not due to compensation from Thrα and similar across other TH targets, including *Thrsp*, *Fasn*, and *Chrebp* (Supplemental Figures S1A, B, http://links.lww.com/HC9/C319). In addition, there was no difference in thyroid-stimulating hormone (TSH) or tetraiodothyronine (T4) between control and L-TRBKO mice, indicating that hepatic *Thr*β*1* does not contribute to systemic thyroid hormone levels at baseline (Figure 2B). Systemic glucose homeostasis was also unaffected, as demonstrated by unchanged fasting glucose and insulin levels, and an i.p.GTT did not differ between control and L-TRBKO mice (Figure 2C). To assess the role of Thrβ1 in the liver’s response to insulin, activation of the hepatic insulin signaling pathway was measured by phosphorylation of Akt in the liver.^36^ Akt phosphorylation did not differ between control and L-TRBKO mice (Figure 2D).^36^ These results indicate that hepatic Thrβ1 does not affect circulating thyroid hormone levels, systemic glucose metabolism, or activation of the hepatic insulin signaling pathway in euthyroid chow-fed mice. These findings were replicated in female mice (Supplemental Figures S2A–D, http://links.lww.com/HC9/C320).

### Hepatic Thrβ1 is not required to improve insulin sensitivity in hypothyroidism

**FIGURE 3:**
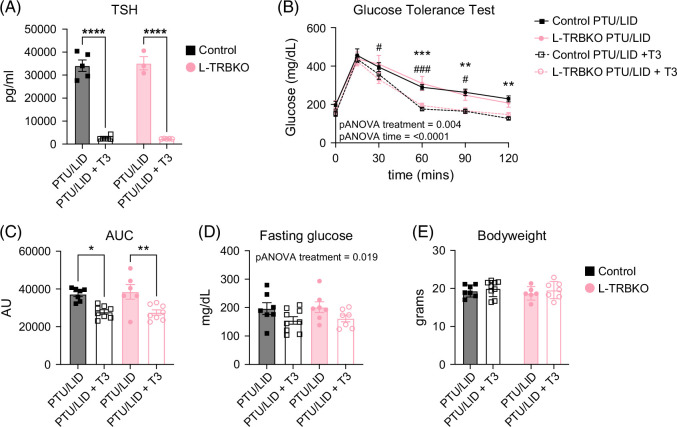
Hypothyroidism impairs systemic glucose tolerance independently of liver thyroid hormone receptor β1. (A) Serum thyroid-stimulating hormone (TSH, or thyrotropin) in male L-TRBKO mice and controls on propylthiouracil/low iodine diet (PTU/LID) treated with T3 (daily i.p. injections with 2 μg T3/100 g body weight for 4 consecutive days) or equivalent volumes of sterile saline. (B) Systemic glucose concentrations during a glucose tolerance test (GTT) after an overnight fast using i.p. injection with 2 g glucose/kg body weight in male L-TRBKO mice and controls on PTU/LID with and without T3 treatment. (C) Area under the curve (AUC) of systemic glucose concentrations during ipGTT. (D) Fasting blood glucose and blood insulin levels in male L-TRBKO mice and controls on PTU/LID with and without T3 treatment. (E) Body weight of male L-TRBKO and control mice on PTU/LID with and without T3 treatment. Data is shown as mean ± SEM. * denotes Control PTU/LID versus Control PTU/LID + T3, # denotes L-TRBKO versus L-TRBKO + T3. *, #*p*<0.05, **p<0.01, ***, ###*p*<0.001, ****p<0.0001. N=3–6 for TSH, N=6–8 for GTT. Abbreviations: ipGTT, intraperitoneal glucose tolerance test; L-TRBKO, liver-specific THRβ knock-out; T3, triiodothyronine.

We next sought to answer how the hypothyroid state impacts systemic glucose tolerance in the absence of hepatic Thrβ1. Male mice were treated with a propylthiouracil/low iodine diet (PTU/LID) to induce hypothyroidism, after which they were dosed with either PBS or T3 to induce hyperthyroidism for 4 days (Figure 1A). The PTU/LID diet resulted in severe hypothyroidism, as evidenced by elevated TSH levels in the mice treated with PTU/LID + PBS, whereas an expected reduction in TSH was seen in the mice treated with PTU/LID + T3 (Figure 3A). The change in TSH levels did not differ between control and L-TRBKO mice. Hypothyroidism led to increased IR in both control and L-TRBKO mice as evidenced by higher glucose levels during an i.p. glucose tolerance test (GTT) (Figures 3B, C). Although T3 treatment did affect fasting glucose overall, there were no significant differences between the individual groups (Figure 3D). The IR was completely reversed to the same degree in both genotypes after T3 treatment, indicating that hepatic Thrβ1 is not required for reversal of IR in the hypothyroid state (Figures 3B, C). Finally, body weight was not impacted by the deletion of hepatic Thrβ1, and it was unchanged by T3 treatment (Figure 3E). These results show that changes in thyroid status profoundly affect systemic insulin sensitivity, and that these changes occur independently of liver Thrβ1.

### HFD leads to weight gain and insulin resistance in control and L-TRBKO mice

**FIGURE 4:**
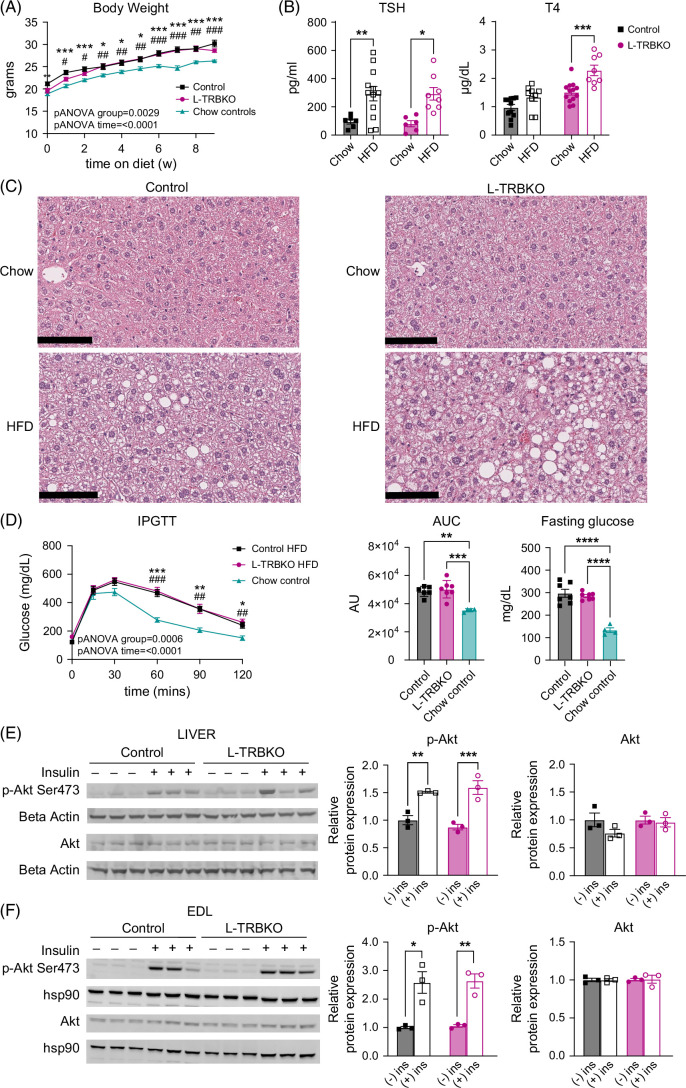
A high-fat diet causes insulin resistance independently of liver thyroid hormone receptor β1. (A) Body weight of male L-TRBKO and control mice on high-fat diet (HFD) and chow-fed controls. (B) Serum TSH and T4 concentrations in L-TRBKO mice and controls on HFD or normal chow diet. (C) Representative images of H&E staining of mouse liver from L-TRBKO mice and controls on HFD or normal chow diet. (D) Systemic glucose concentrations during an i.p. glucose tolerance test (ipGTT) after an overnight fast in male L-TRBKO mice and controls on HFD or normal chow diet. Area under the curve (AUC) for systemic glucose concentrations during ipGTT and fasting glucose at the start of the ipGTT is also shown. (E, F) L-TRBKO and control mice on HFD were injected intraperitoneally with 1 IU/kg human insulin 10 minutes before euthanasia. Phosphorylation of Akt in the liver and skeletal muscle (EDL) was measured using a western blot to assess activation of the insulin signaling pathway. Data are shown as mean ± SEM. * denotes Control versus chow controls. # denotes L-TRBKO vs. chow controls. *, #*p*<0.05, **, ##*p*<0.01, ***, ###*p*<0.001, ****, ####*p*<0.0001. N=6–12 per group. Abbreviations: H&E, hematoxylin and eosin; L-TRBKO, liver-specific THRβ knock-out; T4, tetraiodothyronine; TSH, thyroid-stimulating hormone.

A separate cohort of male control and L-TRBKO male mice was treated with an HFD to induce body weight gain and IR. Similar levels of weight gain occurred in both control and L-TRBKO mice while on the HFD, which was higher than that of normal chow diet (NCD) fed controls (Figure 4A). HFD resulted in changes in circulating thyroid hormones compared with NCD controls. TSH was elevated in control and L-TRBKO mice treated with HFD, a finding that has been described in rats.^37^ In L-TRBKO mice, a rise in circulating T4 was seen in those on an HFD, but this was not seen in controls (Figure 4B). Hepatic steatosis was seen in both control and L-TRBKO mice treated with an HFD (Figure 4C). Mice on HFD developed IR and elevated fasting glucose levels as compared with mice on a NCD (Figure 4D). The effect of HFD on systemic IR and fasting glucose was similar between L-TRBKO mice and controls. We repeated Akt/p-Akt western blots, which showed p-Akt signaling in both control and L-TRBKO mice was preserved despite the IR in the liver (Figure 4E), which appears somewhat blunted compared with NCD-fed mice (Figure 2D). This is likely from systemic IR requiring higher doses of insulin for phosphorylation/signaling to occur. We also assessed skeletal muscle (EDL) Akt-p-AKT, which shows similar patterns to liver upon insulin treatment (Figure 4F). Overall, these data show that systemic IR due to HFD develops independently of hepatic Thrβ1, and there is diminished hepatic insulin signaling in both control and L-TRBKO mice. This suggests that hepatic Thrβ1 is not required for insulin-mediated p-Akt signaling in the liver.

### A single dose of T3 reverses insulin resistance in male mice treated with an HFD

**FIGURE 5:**
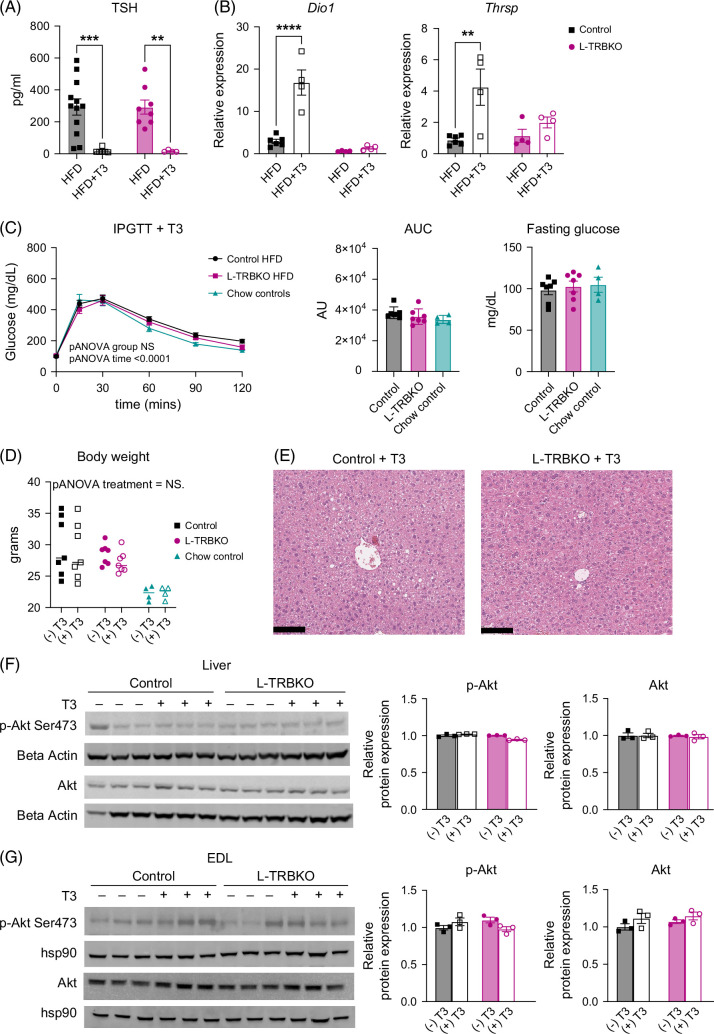
A single injection of T3 normalizes systemic glucose tolerance in mice with insulin resistance due to a high-fat diet (HFD). (A) Serum TSH concentrations in L-TRBKO mice and controls on HFD with and without T3 treatment (daily i.p. injections of 2 μg T3/100 g body weight or equivalent volume of sterile saline for 2 consecutive days). (B) Liver mRNA expression levels of *Thrb1* and *Dio1*, 2 positively regulated T3 target genes, in L-TRBKO mice and controls on HFD with and without T3 treatment (daily i.p. injections of 2 μg T3/100 g body weight or equivalent volume of sterile saline for 2 consecutive days). (C) Systemic glucose concentrations during an i.p. glucose tolerance test (ipGTT) in male L-TRBKO mice and controls on HFD or normal chow diet. All animals were treated with a single i.p. injection 2 μg T3/100 g body weight 17 hours before the ipGTT and fasted overnight. Area under the curve (AUC) for systemic glucose concentrations during the ipGTT and fasting glucose at the start of the ipGTT are also shown. (D) Body weight of male L-TRBKO and control mice on HFD and chow-fed controls treated with daily i.p. injections of T3 or saline as above. (E) Representative images of H&E staining of the liver from L-TRBKO mice and controls on HFD treated with T3 (daily i.p. injections of 2 μg T3/100 g body weight or equivalent volume of sterile saline for 2 consecutive days). (F) Phosphorylation of Akt in the liver was measured using a western blot to assess activation of the insulin signaling pathway in L-TRBKO and control mice on HFD with and without T3 treatment (daily i.p. injections of T3 or saline as above). Data are shown as mean ± SEM. *p<0.05, **p<0.01, ***p<0.001, and ****p<0.0001. N=4–12 per group. Abbreviations: H&E, hematoxylin and eosin; L-TRBKO, liver-specific THRβ knock-out; mRNA, messenger RNA; T3, triiodothyronine; TSH, thyroid-stimulating hormone.

To better understand the role of TH in hepatic insulin signaling during IR, we treated control and L-TRBKO mice on an HFD with T3. Mice received a single dose of T3 before undergoing an i.p. GTT. They then received a second dose of T3, followed by euthanasia the following day to allow for tissues to be collected in the fasted state (Figure 1B). We saw an appropriate reduction in TSH after T3 treatment (Figure 5A) in each group. This was associated with an increase in the Thrβ1-regulated TH target genes *Dio1* and *Thrsp* in control mice treated with T3 but not in L-TRBKO mice (Figure 5B). A single dose of T3 resulted in complete normalization of glucose levels during an i.p. GTT to the level of NCD controls in both L-TRBKO and control mice (Figure 5C). This improvement in IR was not secondary to any reduction in body weight, as both control and L-TRBKO mice had similar and significantly higher body weight compared with NCD controls (Figure 5D). Two doses of T3 treatment also improved the hepatic steatosis associated with an HFD in both control and L-TRBKO mice, as seen by representative H&E images (Figure 5E). Akt and p-Akt protein levels were found to be the same between control and L-TRBKO mice in the liver and EDL (Figures 5F, G). This data shows that a single dose of T3 improves systemic IR due to HFD independently of hepatic Thrβ1 and that the phosphorylation of Akt is completely independent of T3. This was not the case in female L-TRBKO mice, who also develop IR on an HFD but do not show normalization of glucose after T3 treatment compared with chow controls (Supplemental Figures S3A–C, http://links.lww.com/HC9/C321), pointing to sex-specific differences underlying IR and glucose regulation.

### T3 treatment increases glucose transporter expression in skeletal muscle

**FIGURE 6:**
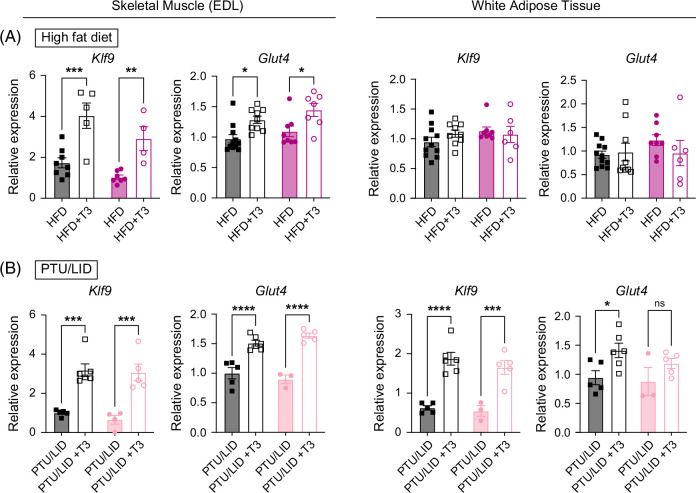
T3 treatment increases glucose transporter expression in skeletal muscle. (A) Skeletal muscle and white adipose tissue mRNA expression levels of *Klf9*, a ubiquitous positively regulated T3 target gene, and *Glut4*, the main glucose transporter in muscle and white adipose tissue, in L-TRBKO mice and controls on high-fat diet (HFD) with and without T3 treatment (daily i.p. injections of 2 μg T3/100 g body weight or equivalent volume of sterile saline for 2 consecutive days). (B) Skeletal muscle and white adipose tissue mRNA expression levels of *Klf9*, a ubiquitous positively regulated T3 target gene, and *Glut4*, the main glucose transporter in muscle and white adipose tissue, in L-TRBKO mice and controls on propylthiouracil/low iodine diet (PTU/LID) rendering them hypothyroid, with and without T3 treatment (daily i.p. injections of T3 or saline as above). Data are shown as mean ± SEM. *p<0.05, **p<0.01, ***p<0.001, and ****p<0.0001. N=3–6 per group. Abbreviations: L-TRBKO, liver-specific THRβ knock-out; mRNA, messenger RNA; T3, triiodothyronine; TSH, thyroid-stimulating hormone.

As the improvement in systemic IR by a single dose of T3 was not mediated via hepatic Thrβ1, we sought to determine whether TH action in other tissues was responsible for the improvement in glucose tolerance. White adipose tissue (WAT) and skeletal muscle play important roles in insulin tolerance and are key thyroid hormone target tissues. Short-term T3 treatment resulted in a robust increase in *Klf9*, a ubiquitous thyroid hormone target gene, in skeletal muscle but not in WAT (Figure 6A). T3 treatment also increased expression of *Glut4* in skeletal muscle, but not in WAT. Glut4 is the primary glucose transporter and regulator of glucose uptake in both skeletal muscle and WAT. The increase in skeletal muscle *Glut4* expression in response to T3 was also observed in mice rendered hypothyroid by PTU/LID and then treated with T3 (Figure 6B). In these hypothyroid male mice, T3 treatment increased *Klf9* expression in both skeletal muscle and WAT, consistent with previous data (Figure 6B). Unlike in the HFD-treated mice, *Glut4* expression in WAT significantly increased following T3 treatment in control animals on PTU/LID, but not in L-TRBKO mice on PTU/LID. These results suggest that the improvement in IR after T3 treatment may be mediated by increased glucose uptake in skeletal muscle. In female mice, there was no increase in either skeletal muscle or WAT *Klf9* or *Glut4* in mice fed a HFD treated with T3, highlighting a potential mechanism of sex-specific differences in response to T3 (Supplemental Figures S3D, E, http://links.lww.com/HC9/C321).

### Hepatic Thrβ1 does not significantly regulate body composition or energy expenditure

**FIGURE 7:**
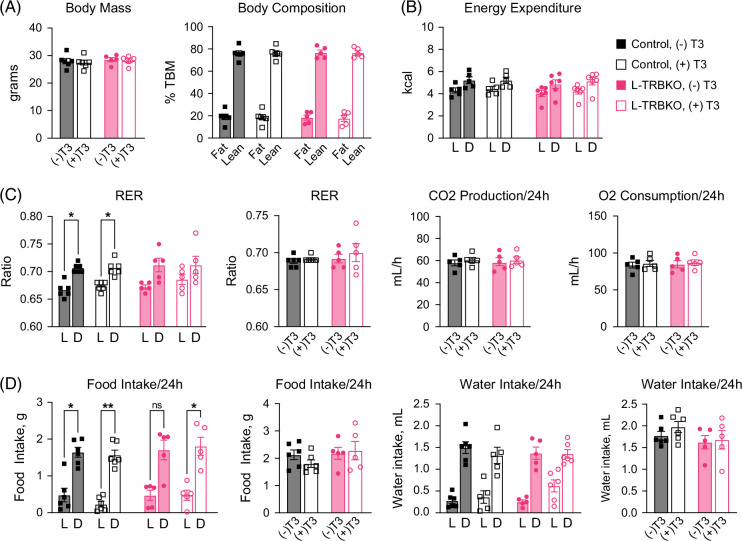
Body composition and energy expenditure are not affected by a lack of liver thyroid hormone receptor β1, nor by T3 treatment. L-TRBKO mice and controls on high-fat diet (HFD) before and after a single dose of T3 treatment (i.p. injection of 2 μg T3/100 g body weight) (A) Body mass, lean mass, and fat mass as measured by EchoMRI. (B) Energy expenditure (EE) for light (L) and dark (D) cycles normalized to lean body mass by analysis of covariance. (C) Respiratory exchange ratio (RER) for light (L) and dark (D) cycles and over 24 hours. CO_2_ production and O_2_ consumption over 24 hours. (D) Food and water intake for light (L) and dark (D) cycles and over 24 hours. Data are shown as mean ± SEM. *p<0.05, **p<0.01, ***p<0.001, and ****p<0.0001. N=5–6 per group. Abbreviations: L-TRBKO, liver-specific THRβ knock-out; T3, triiodothyronine; TSH, thyroid-stimulating hormone.

Given the findings that hepatic Thrβ1 has no role in improving systemic IR, we next sought to determine the role of hepatic Thrβ1 in systemic energy metabolism. Male control and L-TRBKO mice on HFD underwent metabolic phenotyping as previously described.^30^ Following 24 hours of baseline measurements, mice were treated with a single dose of i.p. T3, after which measurements were repeated. Both body mass and body composition, including lean and fat mass, were unchanged between control and L-TRBKO mice treated with and without T3 (Figure 7A). Similarly, energy expenditure was no different between these groups (Figure 7B). While the respiratory exchange ratio (RER) was significantly higher in control mice treated with and without T3 in the dark versus light cycle, this was not the case in L-TRBKO mice. However, over a 24-hour period, there was no difference in RER among groups (Figure 7C). Neither food intake nor water intake was different between control and L-TRBKO mice (Figure 7D). Overall, hepatic Thrβ1 is not required to regulate metabolism in response to a single dose of T3. The improvement in systemic glucose tolerance due to a single T3 injection in insulin-resistant mice is not mediated via body composition, energy expenditure, or RER.

### Insulin signaling is conserved in human hepatocytes lacking hepatic THRβ1

**FIGURE 8:**
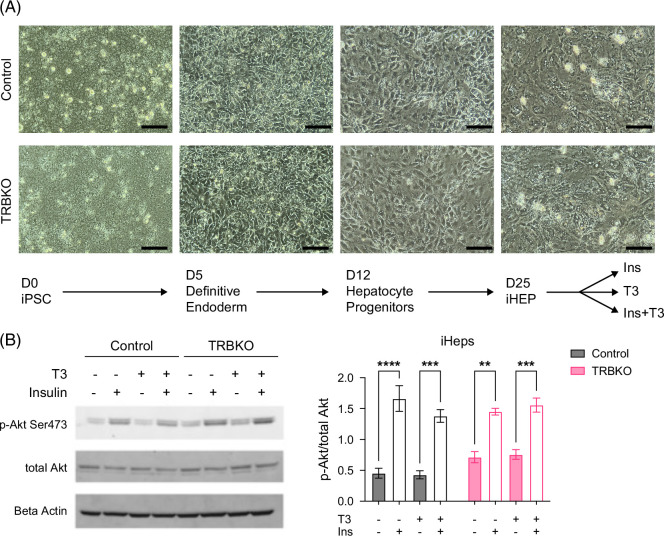
Insulin signaling is conserved in human iPSC-derived hepatocytes lacking THRβ1. (A) Hepatocyte-like cells (iHeps) derived from a WT and TRBKO iPSC line were morphologically similar during differentiation from iPSC into iHeps. Following differentiation into iHeps, cells were treated with insulin (Ins), T3, insulin + T3 (Ins+T3), or vehicle control. (B) Phosphorylation of Akt in iHeps was measured using a western blot to assess activation of the insulin signaling pathway following treatment with a combination of T3, insulin, and/or vehicle control as indicated. Left panel: representative image from 1 experiment, right panel: quantification of 3 independent differentiations. Data shown as mean ± SEM. N=3 biological replicates per group from separate differentiations. Abbreviations: iHeps, iPSCs induced to human hepatocytes; iPSC, inducible pluripotent stem cell; T3, triiodothyronine; TRBKO, THRβ knock-out; WT, wild type.

To assess the role of hepatic THRβ1 in human hepatocytes, we differentiated human iPSCs to hepatocyte-like cells (iHeps) using a previously established protocol.^35^ We have shown that these iHeps are T3 responsive with conserved T3 signaling pathways.^28^ In addition, these iHeps are functional, and THRβ1 is critical for stimulating both T3 induced gluconeogenesis and maintaining a balance of lipid pathways in hepatocytes.^33^ To interrogate THRβ1 signaling in human cells, we previously applied CRISPR/Cas9 to wild-type (WT) iPSCs to delete exon 8 of THRβ, a part of the ligand-binding domain, resulting in a loss of THRβ gene expression.^33^ A lack of *THR*β*1* does not affect iHep differentiation or morphology (Figure 8A).^33^ Following the standard differentiation protocol, D25 WT and TRBKO iHeps were incubated in washout medium for 24 hours to avoid activation of Akt by other components of the medium. iHeps were then incubated with or without 100 M T3 in washout medium for another 24 hours, followed by 10 minutes of insulin treatment or vehicle control before cell harvest. iHeps with and without *THR*β*1* were able to activate the Akt signaling pathway in response to insulin to a similar degree; this was not affected by T3 treatment. T3 treatment alone did not induce Akt phosphorylation (Figure 8B). This indicates that T3 does not affect the insulin signaling pathway in human hepatocytes and that THRβ1 is not required for insulin signaling in human hepatocytes.

## DISCUSSION

Here, we show that hepatocyte-specific THRβ1 does not affect systemic glucose tolerance, which has not previously been explored. Both HFD and hypothyroidism result in systemic insulin resistance, independently of hepatocyte THRβ1. In addition, we show that short-term T3 treatment normalizes systemic glucose tolerance in insulin-resistant mice and that these effects are independent of hepatic THRβ1. This is somewhat unexpected given the clinical association between thyroid disease and impaired glucose tolerance and the known role of TH in hepatic glycogenolysis and gluconeogenesis.^18^^,^^38^

The first THRβ agonist was recently approved for the treatment of liver fibrosis, and several other THRβ agonists are currently under evaluation for the treatment of metabolic diseases, which are intricately linked to glucose metabolism and IR.^13^^,^^14^^,^^39^ Clinical and pre-clinical studies have yielded discrepant results regarding the effects of THRβ agonists on glucose levels and insulin sensitivity.^13^^,^^24^ Given that up to half of patients with MASLD have diabetes, it is critical to understand how hepatic THRβ impacts these parameters. In ob/ob mice, the THRβ agonists GC-1 and KB2115 improve hepatic triglyceride levels and overall hepatic steatosis without affecting insulin sensitivity.^40^ Other studies have reported a worsening of insulin sensitivity and the development of fasting hyperglycemia after treatment with GC-1 in rodents.^24^ The available clinical data from human trials do not show a clear effect on systemic glucose tolerance, although the reported data so far are minimal.^13^^,^^39^ Furthermore, the precise mechanisms behind the beneficial clinical effects of these THRβ agonists have proved less clear than previously thought, as illustrated by recent data from animal models showing that hepatic Thrβ receptor levels do not impact MASH development or progression.^41^

The induction of hepatic gluconeogenesis is thought to contribute to hyperglycemia and IR observed in rodents following treatment with THRβ agonists.^16^^,^^24^ The THRβ1-dependent induction of gluconeogenesis by thyroid hormone in hepatocytes is well established.^16^^,^^38^ T3 treatment increases SIRT1-dependent deacetylation of FOXO1, a key transcription factor for gluconeogenesis.^16^^,^^17^^,^^42^ This activation of FOXO1, which leads to increased expression of gluconeogenic genes in the liver, is mediated via THRβ1.^16^ We have previously shown that iHeps that lack THRβ1 demonstrate impaired production of glucose from pyruvate and lactate.^33^ Interestingly, the data shown here suggest that this impairment of hepatocyte gluconeogenesis due to a lack of THRβ1 does not affect systemic glucose tolerance as measured by i.p. GTT either at baseline or during IR. Although thyroid hormone has been implicated as an important factor in glucose homeostasis and insulin signaling based on clinical and rodent data, the specific mechanisms behind these effects remain largely elusive. Our data show that induction of hepatic insulin signaling, as measured through the phosphorylation of Akt, can occur independently of THRβ1 in both human and mouse hepatocytes and is not affected by T3 treatment.

The thyroid hormone acts independently of hepatocyte-specific THRβ1 to improve systemic IR in our model. The effect of T3 appears to be mediated, at least in part, via upregulation of the key glucose transporter GLUT4 in skeletal muscle. This induction of GLUT4 by thyroid hormone in skeletal muscle has previously been demonstrated in hyperthyroid mice and was accompanied by an increase in insulin receptor signaling in skeletal muscle.^43^ Our results suggest that even a single dose of T3 can induce this mechanism and normalize systemic glucose tolerance in male mice. This is further supported by the lack of a similar correction in female mice, where thyroid hormone treatment fails to induce Glut4 expression.

Another extrahepatic mechanism contributing to the improvement of systemic glucose tolerance by T3 is the induction of glucagon-like peptide 1 (GLP-1) through repression of intestinal farnesoid-X receptor (FXR) signaling due to changes in bile acid composition.^44^ Like our findings, this study found that 5 days of T3 treatment results in improved glucose tolerance in hypothyroid mice after both oral and i.p. glucose challenge. In mice that received an oral glucose challenge, the observed improvement in glucose tolerance after T3 treatment is accompanied by an increase in circulating GLP-1. The authors elegantly show that this effect is mediated via hepatic THRβ, which downregulates *Cyp8b1* in the liver, resulting in changes in bile acid composition that repress intestinal FXR-signaling, thereby potentiating GLP-1 production and insulin secretion.^44^ As we did not treat mice with an oral glucose challenge, but with i.p. glucose challenge, we cannot exclude that there may be changes in glucose tolerance in our model following an oral glucose challenge. Our results show that the T3-mediated improvement in glucose tolerance following i.p. glucose challenge does not require hepatic THRβ1, suggesting that the effects of hepatic THRβ1 on GLP-1 secretion observed by Yan et al. are not involved here, as i.p. glucose does not elicit an incretin response.

Classically, T3’s actions have been described in the context of its binding to the THR, thus promoting thyroid hormone-responsive gene expression or THR-mediated non-canonical signaling. However, non-genomic, extra-nuclear actions of T3 independent of the THR have been described and include acting on the Na+/K+ ATPase in hepatocytes and induction of hepatic mitochondrial respiration.^45^^,^^46^ Given mitochondrial dysregulation has been implicated in the development of MASLD,^47^ it is reasonable to hypothesize that T3 actions on mitochondria could impact hepatic function. While we have deleted THRβ1 in hepatocytes, we cannot exclude the induction of non-genomic effects of T3 that do not require THR signaling within hepatocytes.

There are well-documented sex-specific differences in the development and progression of MASLD, insulin resistance, and type 2 diabetes in human and animal studies. Estrogen has been suggested to exert protective metabolic effects, while androgens may promote MASLD development.^48^ Consistent with this, sex-dependent regulation of inflammatory pathways has been reported. For example, adipose IRF5 correlates with higher fasting glucose levels, metabolic dysfunction, insulin, and hemoglobin A1c levels in males but not females.^49^ Moreover, recent human data indicate that higher insulin resistance in women with MASLD is associated with increased mortality.^50^ In line with these observations, our data demonstrate a sex-specific response to TH treatment, as short-term T3 normalized blood glucose only in male mice. This suggests that TH-mediated metabolic effects are modulated by sex-specific pathways and differences in tissue-sensitivity that should be further investigated. Historically, published data have frequently included only male animals, meaning that the literature on sex-specific effects of TH is extremely limited. Our data confirm the importance of studying both sexes in animal models.

Taken together, our data indicate that T3 plays a crucial role in whole-body glucose metabolism; however, this effect is independent of liver THRβ1 and is mediated via extrahepatic mechanisms. There is also a sex-specific difference in response to T3 between male and female mice with IR that warrants further evaluation. As THRβ agonists continue to be developed and used for the treatment of MASLD, it is critical to delineate their mechanism of action at the molecular level.

## Supplementary Material
