# Supplementary material for: Thyroid hormone acts independently of the thyroid hormone receptor beta in hepatocytes to improve systemic insulin sensitivity
Source: Hepatol Commun. 2026 May 8;10(6):e0937. doi: 10.1097/HC9.0000000000000937 (PMC13218675; doi:10.1097/HC9.0000000000000937)

Supplemental Figure 1: Loss of Thrβ1 does not significantly alter TH responsive genes in a euthyroid state and there is no Thrα compensation. Liver mRNA expression levels of *Thrα* (A) and *Thrsp, Fasn* and *Chrebp* (B) in male L-TRBKO mice and controls. Data is shown as mean +/- SEM. N=5-7 per group.

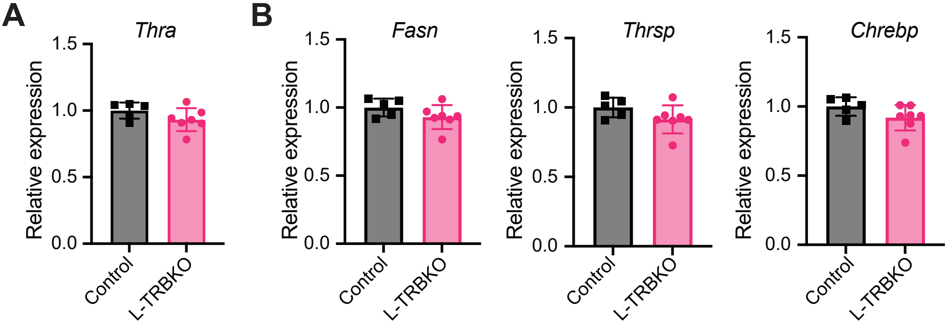

Supplement: Supplementary file 1 [file hc9-10-e0937-s001.pdf]
