# Supplementary material for: Thyroid hormone acts independently of the thyroid hormone receptor beta in hepatocytes to improve systemic insulin sensitivity
Source: Hepatol Commun. 2026 May 8;10(6):e0937. doi: 10.1097/HC9.0000000000000937 (PMC13218675; doi:10.1097/HC9.0000000000000937)

Supplemental Figure 2: Female mice respond similarly to euthyroid male mice lacking hepatic Thrβ. (A) Liver mRNA expression levels of *Thrb1* and *Dio1* in female L-TRBKO mice and controls. (B) Circulating thyroid stimulating hormone (TSH, or thyrotropin) in female L-TRBKO mice and controls. (C) Fasting blood glucose and blood insulin levels in female L-TRBKO mice and controls. (D) ipGTT in female L-TRBKO mice and controls after an overnight fast. (D) Female L-TRBKO and control mice were injected intraperitoneally with 1 IU/kg human insulin 10 minutes prior to euthanasia. Phosphorylation of Akt in the liver was measured using Western blot to assess activation of the insulin signaling pathway. Data is shown as mean +/- SEM. * = p<0.05, ** = p< 0.01, *** = p<0.001. N=4-7 per group.

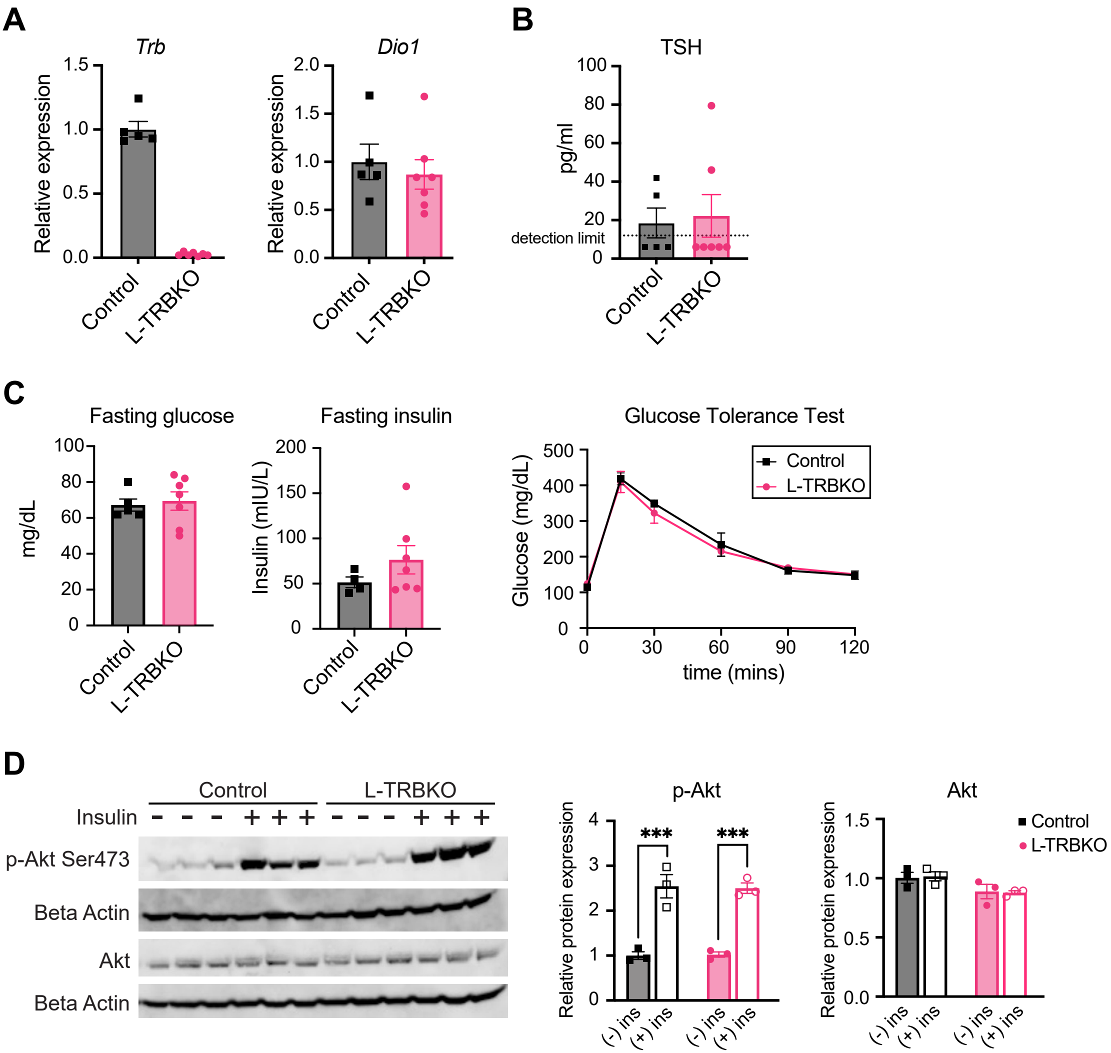

Supplement: Supplementary file 2 [file hc9-10-e0937-s002.pdf]
