# Supplementary material for: Thyroid hormone acts independently of the thyroid hormone receptor beta in hepatocytes to improve systemic insulin sensitivity
Source: Hepatol Commun. 2026 May 8;10(6):e0937. doi: 10.1097/HC9.0000000000000937 (PMC13218675; doi:10.1097/HC9.0000000000000937)

Supplemental Figure 3: Female mice have no improvement in glucose levels with T3 treatment. (A) Bodyweight of female L-TRBKO and control mice on high fat diet (HFD) and chow fed controls. (B) ipGTT in female L-TRBKO and control mice on a HFD. (C) ipGTT in female L-TRBKO and control mice on a HFD treated with T3. (D) Skeletal muscle mRNA expression of *Klf9* and *Glut4*. (E) WAT mRNA expression of *Klf9* and *Glut4*. Data is shown as mean +/- SEM. * denotes Control vs. chow controls. # denotes L-TRBKO vs. chow controls. *,# = p<0.05, **, ## = p< 0.01, ***, ### = p<0.001, ****, ####= p<0.0001. N=4-9 per group.

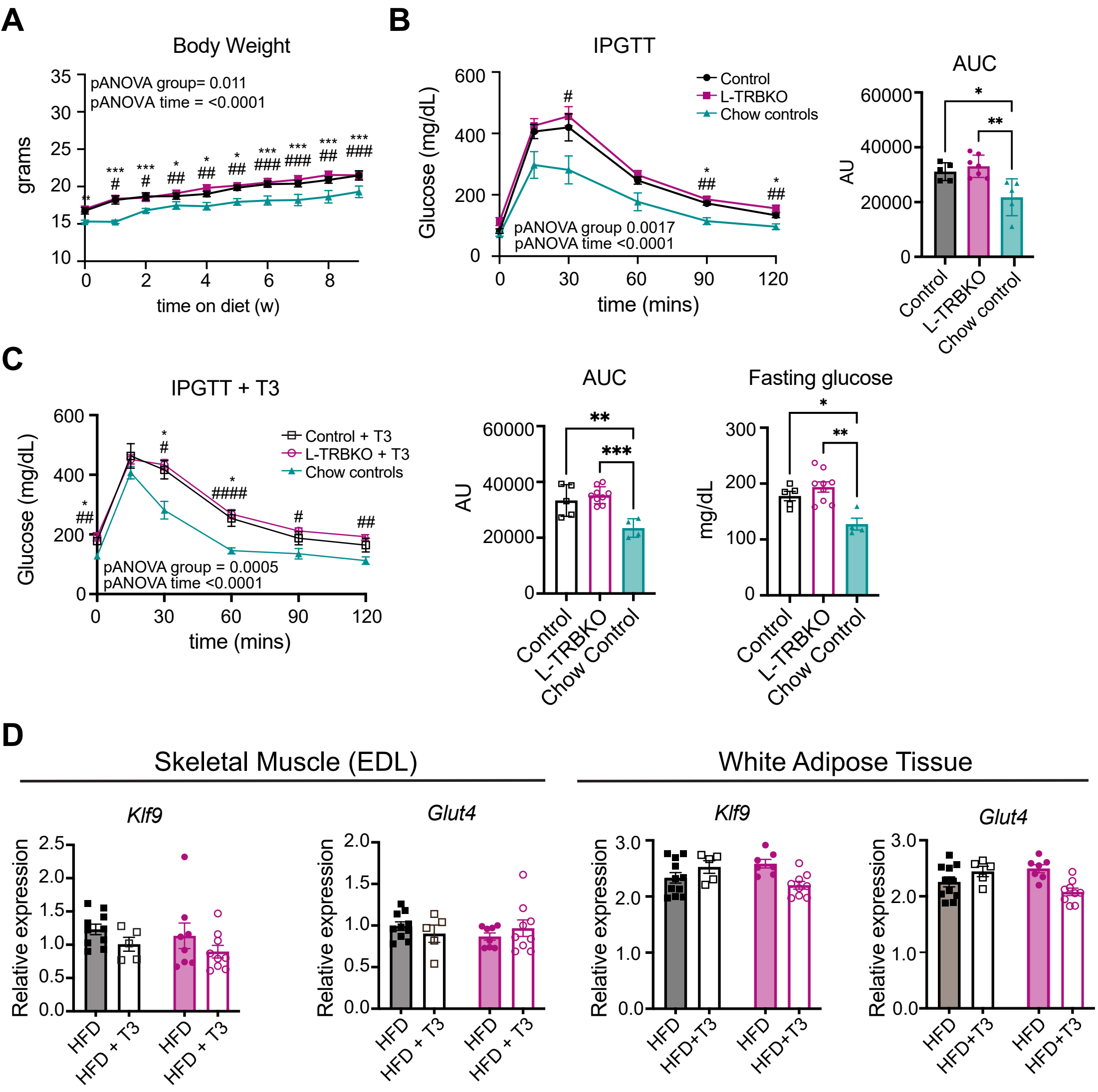

Supplement: Supplementary file 3 [file hc9-10-e0937-s003.pdf]
